# Longitudinal Measurement Invariance of the ASEBA Youth/Adult Self-Reports Across the Transition From Adolescence to Adulthood

**DOI:** 10.1177/10731911241245875

**Published:** 2024-04-18

**Authors:** Daniel P. Moriarity, Naoise Mac Giollabhui, Dener Cardoso Melo, Catharina Hartman

**Affiliations:** 1 University of California, Los Angeles, USA; 2 Massachusetts General Hospital and Harvard Medical School, Boston, USA; 3 Interdisciplinary Center Psychopathology and Emotion Regulation (ICPE), Department of Psychiatry, University Medical Center Groningen, University of Groningen, The Netherlands

**Keywords:** measurement, measurement invariance, longitudinal, adolescent, adult, developmental, psychopathology

## Abstract

The ability to quantify within-person changes in mental health is central to the mission of clinical psychology. Typically, this is done using total or mean scores on symptom measures; however, this approach assumes that measures quantify the same construct, the same way, each time the measure is completed. Without this quality, termed longitudinal measurement invariance, an observed difference between timepoints might be partially attributable to changing measurement properties rather than changes in comparable symptom measurements. This concern is amplified in research using different forms of a measure across developmental periods due to potential differences in reporting styles, item-wording, and developmental context. This study provides the strongest support for the longitudinal measurement invariance of the Anxiety Scale, Depression/Affective Problems: Cognitive Subscale, and the Attention Deficit Hyperactivity Disorder (ADHD) Scale; moderate support for the Depression/Affective Problems Scale and the Somatic Scale, and poor support for the Depression/Affective Problems: Somatic Symptoms Subscale of the Dutch Achenbach System of Empirically Based Assessment Youth Self-Report and Adult Self-Report in a sample of 1,309 individuals (*N* = 1,090 population-based, *N* = 219 clinic-based/referred to an outpatient clinic before age 11 years) across six waves of data (mean ages = 11 years at Wave 1 and 26 years at Wave 6).

Central to psychology’s mission to examine mechanisms and treatments for psychopathology is the ability to measure change in symptoms over time. Studies typically quantify change via increases or decreases in total scores on self-report measures; however, this assumes that the total score quantifies symptoms the same way at each time point. For example, change score approaches assume that a score of 13 at baseline is comparable to a score of 13 at posttreatment (i.e., is largely compromised by the same symptom profile and identical factor structure). The ability for a measure to quantify the same construct, the same way, across different times points is referred to as *longitudinal measurement invariance.*

The three most commonly assessed types of measurement invariance are configural, metric (i.e., “weak”), and scalar (i.e., “strong”; for a more detailed review on measurement invariance, see Putnick & Bornstein, 2016). We provide a brief conceptual overview here, with more technical information available in the Methods section. Configural invariance refers to the equivalence of model form (i.e., which items load onto which latent constructs). Metric invariance refers to equality of factor loadings. Scalar invariance refers to equality of item intercepts (i.e., the average response to an item when the associated latent score is zero). For visualization of these measurement invariance types, see Figure 1. Without measurement invariance, a questionnaire does not measure a construct the same way across different time points, precluding mean comparison of scores to evaluate change in the underlying construct. A nonpsychological example would be to consider if you weighed yourself on a scale at home today and re-weighed yourself using the same scale from the moon tomorrow. The subject and measurement tool are constant, but the underlying measurement properties change overtime in a way that invalidates direct comparison of the two measurements.

**Figure 1.**
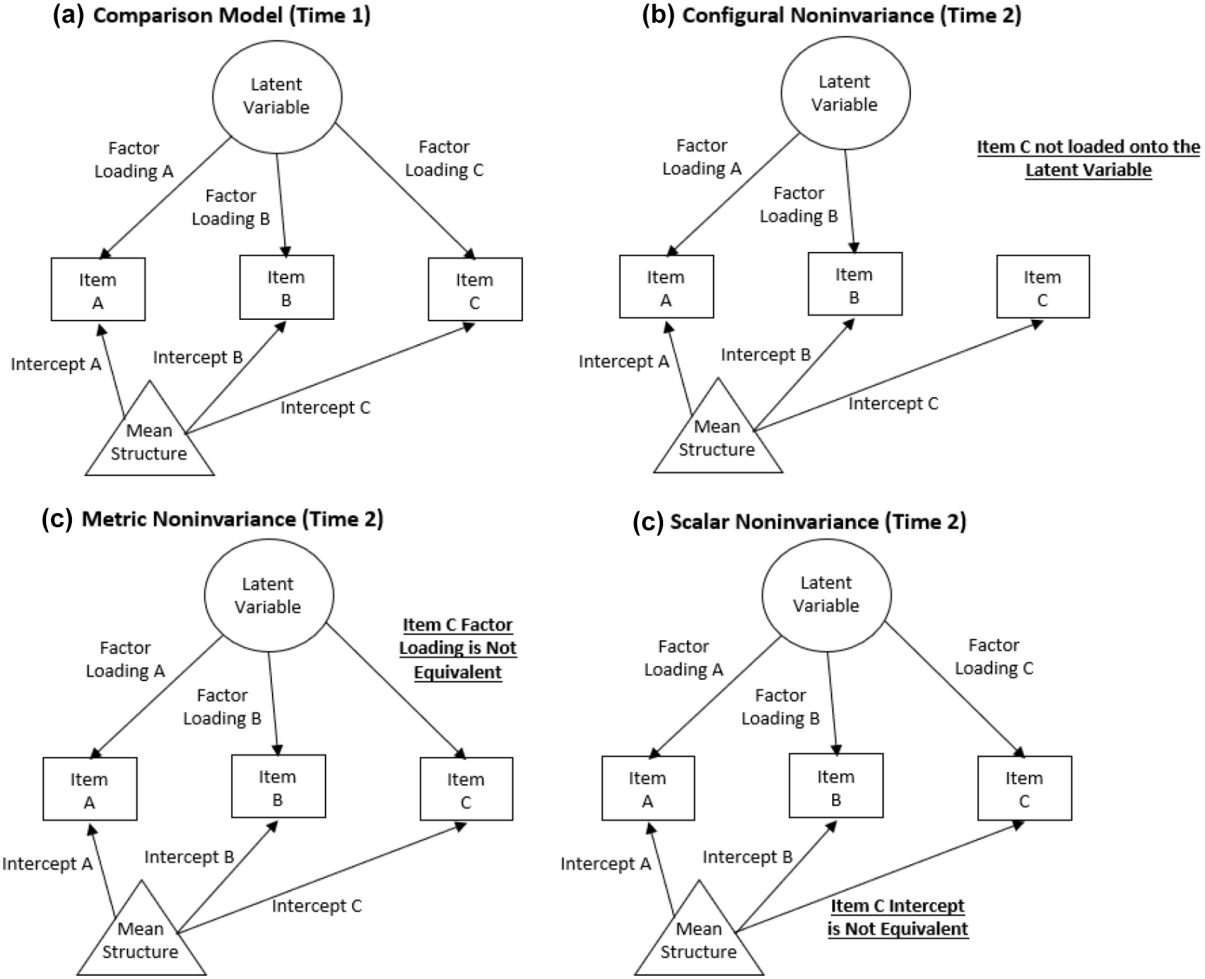
Visualization of Measurement Invariance Types Illustrated by the Measurement Properties of Item C. *Note*. Focal differences associated with the specified type of invariance are highlighted by a bolded and underlined statement.

This concern is amplified when different measures are used to assess the same construct at different time points in a longitudinal study. For example, the TRacking Adolescents’ Individual Lives Survey (TRAILS) is a large prospective cohort following 11-year olds and reassessing them every 2 to 3 years. At the onset of TRAILS, participants completed the Youth Self-Report (YSR; Achenbach, 1991) from the Achenbach System of Empirically Based Assessment (ASEBA, a comprehensive set of assessments designed to assess adaptive and maladaptive functioning) to assess youth psychological health. The YSR includes 112 items and has been disaggregated into several different factor structures based on researcher/clinician needs. The TRAILS data documentation provides two strategies: syndromes (comprised of 11 scales) or *Diagnostic and Statistical Manual of Mental Disorders* (*DSM*)-oriented scales (comprised of six scales). Given that TRAILS waves have been completed during childhood, adolescence, and adulthood, all original participants “aged out” of the YSR by Wave 4 and shifted to completing the more developmentally appropriate Adult Self-Report (ASR; Achenbach & Rescorla, 2003) of the ASEBA system. However, this change in measures, in addition to potential developmental changes in both symptom-reporting by increasingly mature individuals and age-related differences in the latent construct, could be a source of measurement *non*invariance.

Given the size of the ASR/YSR, the few investigations into their longitudinal measurement invariance have tested select subscales to maintain computational brevity. For example, Barzeva et al. (2019) found that the social withdrawal scales were measurement invariant in people measured four times in the TRAILS study using both the YSR and ASR. Research from the Netherlands Twin Registry (Abdellaoui et al., 2012) found that the ASR Thought Problems Subscale was measurement invariant across three age groups (12–18, 19–27, and 28–59 years). However, only one time point per participant was used in this analysis, so longitudinal measurement invariance *within people* was not tested, just *between different age groups*, thus these results only measure between-person differences (e.g., potentially cohort effects) instead of testing measurement invariance across time within individuals. The only study we found testing age-related measurement invariance of the entire eight factor model used a similar age-stratification technique—supporting measurement invariance of the ASR between two age groups (18–35 vs. 36–59 years; Guerrero et al., 2020). Thus, while preliminary evidence supports longitudinal measurement invariance of the YSR/ASR to the extent they have been investigated, much more work is needed. Specifically, (a) additional subscales of the YSR/ASR must be examined (ideally in the same sample), (b) longitudinal measurement noninvariance (vs. age group measurement invariance) must be evaluated to facilitate change-in-symptom research, and (c) measurement invariance of symptoms in individuals transitioning between the YSR and ASR should be investigated to determine the appropriateness of using both in longitudinal research.

The theoretical and clinical utility of a large, longitudinal dataset such as TRAILS for garnering developmental insight through adolescence and across the transition to adulthood is immense, if the foundational psychometric work is done to inform future longitudinal modeling and data collection. Given its widespread use in TRAILS and other studies, the present investigation evaluated the longitudinal measurement invariance of *DSM* (4th ed.; *DSM-IV*; American Psychiatric Association, 1994) scales that are shared between the YSR and the ASR, i.e., Depression/Affective Problems, Anxiety, Attention Deficit Hyperactivity Disorder (ADHD), and Somatic, using six waves of TRAILS Data. Furthermore, this study also tested the longitudinal measurement invariance of two constituent subscales of the Depression Scale/Affective Problems (cognitive and somatic symptoms) previously identified using TRAILS data (Bosch et al., 2009).

## Method

### Participants

Data were drawn from the TRacking Adolescents’ Individual Lives Survey (TRAILS), a prospective cohort study examining psychosocial development and mental health in youth. Adolescents aged 11 years were recruited and invited to attend regular follow-up assessments every 2 to 3 years. Two separate cohorts were followed by TRAILS—one population-based and another clinic-based (Huisman et al., 2008; Oldehinkel et al., 2015). Adolescents in the population-based cohort were recruited from 135 schools in five municipalities in the north of The Netherlands, including both urban and rural areas. Eligible participants were required to be enrolled in primary school, and of 2,935 youth who met this criterion, 2,230 (76%) provided informed consent from both parent and child to participate. The clinic-based cohort consisted of children referred to a psychiatric outpatient clinic before the age of 11 for a variety of psychiatric and behavioral problems. Cohorts were not analyzed separately because inclusion in the “clinical cohort” was strictly determined by recruitment site, and these two groups did not account for the potentiality of (a) participants in the clinical cohort who, at a later point, had no need of/stopped receiving clinical services and (b) participants in the population cohort who, at a later point, were in need of/received clinical services. This study utilized data from 1,309 participants (*N* = 1,090 population-based, *N* = 219 clinic-based) from Waves 1 to 6 (see Table 1 for descriptives and below for data cleaning details).

**Table 1:** Descriptive Statistics for Variables of Interest (N = 1,309).

Variable and Timepoint	*M*	*SD*	Range
Wave 1			
% Female	56.7%		
Age (years)	11.09	.55	10.01–12.54
SES (z)	.16	.75	−1.73–1.73
Depression/affective problems	4.06	3.21	0–18
Somatic subscale	2.29	1.94	0–11
Cognitive subscale	1.77	1.85	0–11
Anxiety	2.28	1.88	0–10
ADHD	4.36	2.56	0–13
Somatic	3.33	2.32	0–11
Wave 2			
Age (years)	13.39	.59	11.58–14.93
Depression/affective problems	3.80	3.38	0–24
Somatic subscale	2.24	2.00	0–10
Cognitive subscale	1.56	1.93	0–14
Anxiety	2.40	1.93	0–10
ADHD	4.77	2.67	0–14
Somatic	2.23	2.02	0–9
Wave 3			
Age (years)	16.15	.66	14.42–18.33
Depression/affective problems	3.97	3.58	0–24
Somatic subscale	2.55	2.18	0–12
Cognitive subscale	1.42	1.94	0–14
Anxiety	2.13	1.90	0–11
ADHD	4.88	2.77	0–14
Somatic	1.82	1.94	0–10
Wave 4			
Age (years)	18.97	.59	17.98–21.06
Depression/affective problems	4.40	4.40	0–24
Somatic subscale	2.10	2.13	0–10
Cognitive subscale	1.37	1.89	0–12
Anxiety	2.83	2.54	0–14
ADHD	5.91	4.45	0–21
Somatic	1.13	2.09	0–14
Wave 5			
Age (years)	22.13	.66	20.74–24.10
Depression/affective problems	4.63	4.37	0–26
Somatic subscale	2.33	2.10	0–9
Cognitive subscale	1.41	1.83	0–13
Anxiety	2.94	2.59	0–13
ADHD	5.53	4.27	0–22
Somatic	1.89	2.19	0–16
Wave 6			
Age (years)	25.66	.63	24.35–27.82
Depression/affective problems	5.60	4.97	0–26
Somatic subscale	2.69	2.27	0–10
Cognitive subscale	1.64	2.08	0–12
Anxiety	3.61	2.94	0–14
ADHD	5.79	4.31	0–25
Somatic	2.19	2.41	0–15

*Note. z* = z-standardized on whole sample (not analytic sample). ADHD = attention deficit hyperactivity disorder.

### Procedures

In this study, symptoms were measured at each assessment (Waves 1–6) using either the YSR or the ASR (determined by participant age at the time of assessment). Children started the study with the YSR at approximately 11 years old and shifted to the ASR when they turned 16 years old (Wave 4).

### Missing Data Analyses

Participants were removed if they were missing 100% of symptom data at any time point (removing these participants solved some issues with model convergence). Individual analytic datasets were created for each scale to maximize sample size by only removing participants missing data on a given *DSM-IV* scale. This resulted in identical analytic datasets for all analyses (*N* = 1,090 population-based, 219 clinic-based) except for the Depression Somatic Symptoms Subscale (*N* = 1,089 population-based, 219 clinic-based) and Somatic Scale datasets (*N* = 1,074 population-based, 217 clinic-based), which were slightly smaller. Because of the negligible difference in samples, descriptive statistics for the analytic dataset corresponding to the majority of scales.

T-tests and chi-square tests examined whether the analytic sample (*N* = 1,309, 83% population cohort, 17% clinic-based cohort) differed significantly from the entire baseline sample (*N* = 2772, 80% population cohort, 20% clinic-based cohort) based on reported age, gender, socioeconomic status (SES), and depression symptoms. The mean level of SES (indexed by a composite of z-standardized variables, see below for more information) in the analytic sample was higher than in the excluded sample (*t*[2,762] = 12.88, *p* < .001; mean difference = .49 standard deviations). Furthermore, the analytic sample was younger (*t*[2,770] = 2.09, *p* = .035; mean difference = .04 years), had higher anxiety symptoms (*t*[2,675.44] = 3.598, *p* < .001; standardized mean difference = .14), and higher somatic symptoms (*t*[2,692] = 2.687, *p* = .007; mean difference = .10). The analytic sample also differed from the excluded sample in the proportion of females that were retained in the sample, *χ*^2^(1,2772)=84.36, *p* < .001, with fewer males present in the analytic sample (Standardized Residual = −4.6). No differences between the analytic and entire sample in baseline depressive symptoms were reported for the Depression/Affective Problems Scale (*t*[2,716] = 1.453, *p* = .146; standardized mean difference = .06), Cognitive Symptoms Subscale (*t*[2,715] = 1.926, *p* = .054; standardized mean difference = .07), Somatic Symptoms Subscale (*t*[2,714] = .584, *p* = .559; standardized mean difference = .02), or ADHD Scale (*t*[2,717] = .278, *p* = .781; standardized mean difference = .01) of the YSR (note degrees of freedom for symptom measures are slightly different due to different degrees of item-level missingness between scales).

## Measures

### Symptoms

During Waves 1 to 3, symptoms were measured using the YSR (Achenbach, 1991). During Waves 4 to 6, symptoms were measured using the ASR were used during (Achenbach & Rescorla, 2003). Item wording can be found in Table 2. All items were answered using a 3-point Likert-type scale (0–2), with higher endorsements indicating more severe symptoms. For the descriptive (Table 1) and missing data analyses (described above) involving symptom summary statistics, scores were determined by taking the average of the items responded to in the scale in interest and then multiplying by the total number of items in the scale. Symptom summary scores were not calculated for observations with <80% of item-level data.

**Table 2:** The Cognitive and Somatic Subscale Item Wording.

Item		
English	Dutch	Scale
There is very little that I like	Er is heel weinig wat ik leuk vind	Depression/affective problems: Cognitive
I cry a lot	Ik huil veel	Depression/affective problems: Cognitive
I intentionally try to injure myself or attempt suicide	Ik probeer mezelf opzettelijk te verwonden of doe zelfmoordpogingen	Depression/affective problems: Cognitive
I don’t eat as well as I should	Ik eet niet zo goed als zou moeten	Depression/affective problems: Somatic
I feel worthless or inferior	Ik voel me waardeloos of minderwaardig	Depression/affective problems: Cognitive
I feel too much guilt	Ik heb te veel last van schuldgevoel	Depression/affective problems: Cognitive
I feel overtired for no apparent reason	Ik voel me oververmoeid zonder duidelijke reden	Depression/affective problems: Somatic
I sleep more than most of my peers during the day and/or at night	Ik slaap meer dan de meeste van mijn leeftijdgenoten overdag en/of ’s nachts (geef aan):	Depression/affective problems: Somatic
I’m thinking about ending my life	Ik denk erover een eind aan mijn leven te maken	Depression/affective problems: Cognitive
I have trouble sleeping	Ik heb problemen met slapen (geef aan)	Depression/affective problems: Somatic
I don’t have much energy	Ik heb niet veel energie	Depression/affective problems: Somatic
I am unhappy, sad or depressed	Ik ben ongelukkig, verdrietig of gedeprimeerd	Depression/affective problems: Cognitive
*I sleep less than most of my peers	*Ik slaap minder dan de meeste van mijn leeftijdgenoten	*Depression/affective problems: Somatic
□I have trouble making decisions	□Ik heb moeite om beslissingen te nemen	□Depression/affective problems
□I feel like I can’t succeed	□Ik heb het gevoel dat ik niet kan slagen	□Depression/affective problems
I am afraid of certain animals, situations or places	Ik ben bang voor bepaalde dieren, situaties of plaatsen	Anxiety
I am nervous or tense	Ik ben nerveus of gespannen	Anxiety
I’m too anxious or scared	Ik ben te angstig of bang	Anxiety
I often worry	Ik maak me vaak zorgen	Anxiety
*I’m too dependent on adults	*Ik ben te afhankelijk van volwassenen	*Anxiety
*I’m afraid to go to school	Ik ben bang om naar school te gaan	*Anxiety
□Palpitations	□Hartkloppingen	□Anxiety
□I’m worried about my family or relatives	□Ik maak me zorgen over mijn familie of gezin	□Anxiety
□I worry about my future	□Ik maak me zorgen over mijn toekomst	□Anxiety
Pains (no stomachache or headache)	Pijnen (geen buikpijn of hoofdpijn)	Somatic
Headache	Hoofdpijn	Somatic
Nausea	Misselijkheid	Somatic
Eye problems (for which glasses or lenses do not help)	Oogproblemen (waarvoor een bril of lenzen niet helpen)	Somatic
Rash or other skin problems	Huiduitslag of andere huidproblemen	Somatic
Stomach ache	Buikpijn	Somatic
Vomit	Overgeven	Somatic
□I feel dizzy or light-headed	□Ik voel me duizelig of licht in mijn hoofd	□Somatic
□Dead feeling or tingling in body parts	□Dood gevoel of tintelingen in lichaamsdelen	□Somatic
I don’t finish things I need to do	Ik maak dingen die ik moet doen niet af	ADHD
I have difficulty concentrating or keeping my attention on something for long periods of time	Ik heb moeite om me te concentreren, of om lang mijn aandacht ergens bij te houden	ADHD
I am impulsive or do things without thinking	Ik ben impulsief of doe dingen zonder er bij na te denken	ADHD
I have trouble sitting still	Ik heb moeite om stil te zitten	ADHD
*I am inattentive or easily distracted	*Ik ben onoplettend of makkelijk afgeleid	*ADHD
*I talk too much	*Ik praat te veel	*ADHD
*I make more noise than other boys or girls	*Ik maak meer lawaai dan andere jongens of meisjes	*ADHD
□I’m too forgetful	□Ik ben te vergeetachtig	□ADHD
□I often accidentally hurt myself, often get injured accidentally	□Ik bezeer me vaak per ongeluk, raak vaak per ongeluk gewond	□ADHD
□I’m not doing well at my job	□Ik doe het niet goed op mijn werk	□ADHD
□I throw myself into things without thinking about the risks	□Ik stort mij in dingen zonder over de risicos na te denken	□ADHD
□People think I’m chaotic	□Mensen denken dat ik chaotisch ben	□ADHD
□I often lose things	□Ik ben vaak dingen kwijt	□ADHD
□I feel restless	□Ik voel me rusteloos	□ADHD
□I’m too impatient	□Ik ben te ongeduldig	□ADHD
□I don’t pay much attention to details	□Ik let niet goed op details	□ADHD

*Note*. Unless otherwise noted the Dutch wording reflects the ASR version of the items; wording for some YSR items is slightly different to be more developmentally appropriate; the English version of the items is translated from the Dutch version and may differ slightly from the wording in the original ASR or YSR. ADHD = attention deficit hyperactivity disorder. *This item was only available in the YSR (Waves 1–3). □ This item was only available in the ASR (Waves 4–6).

#### Depression/Affective Problems

The YSR Depression/Affective Problems Scale had 13 items (split into a seven item Cognitive Subscale and a six item Somatic Subscale based on item content in previous TRAILS studies [Bosch et al., 2009]). The ASR Depression/Affective Problems Scale had 14 items (split into a seven item Cognitive Subscale and a five item Somatic Subscale based on item content in previous TRAILS studies [Bosch et al., 2009], refer to Table 2 to compare items and wording between measures). The Ω reliability coefficient at Waves 1 and 4 (first wave using the ASR) were .74 and .86 (respectively) for the Depression/Affective Problems Scale, .58 and .72 (respectively) for the Somatic Symptoms Subscale, and .69 and .80 (respectively) for the Cognitive Symptoms Subscale.

#### Anxiety

The YSR Anxiety Scale had six items, and the ASR Anxiety Scale had seven items (refer to Table 2 to compare items and wording between measures). The Ω reliability coefficient at Waves 1 and 4 (first wave using the ASR) were .63 and .78, respectively.

#### Attention Deficit Hyperactivity Disorder

The YSR ADHD Scale had seven items, and the ASR ADHD Scale had 13 items (refer to Table 2 to compare items and wording between measures). The Ω reliability coefficient at Waves 1 and 4 (first wave using the ASR) were .71 and .85, respectively.

#### Somatic

The YSR Somatic Scale had seven items and the ASR Somatic Scale had nine items (refer to Table 2 to compare items and wording between measures). The Ω reliability coefficient at Waves 1 and 4 (first wave using the ASR) were .71 and .83, respectively.

### Sociodemographic Variables

Participant sex was assessed at Wave 1, when participants could respond that they identified as “Female,” which was scored as “0” or “Male,” which was scored as “1.” Age was assessed at all assessments. SES was measured at Wave 1 and Wave 4. SES was estimated using five indicators: family income, maternal educational level, paternal educational level, maternal occupational level, and paternal occupational level using the International Standard Classification of Occupations (Ganzeboom & Treiman, 1996). A composite measure of SES was calculated for the TRAILS cohort based on five *z*-transformed indicators (which has been consistently used in TRAILS), with higher values representing higher SES and a one unit difference representing one standard deviation in difference (Jonker et al., 2017). The composite measure of SES was assessed at Wave 1 and Wave 4 and were highly correlated (*r* = .86) with one another, as previously reported (Mac Giollabhui & Hartman, 2022).

### Statistical Methods

All analyses were conducted in R Version 4.2.2 (R Core Team, 2013). Analyses were conducted in lavaan (Rosseel, 2012). Template code was adapted from https://longitudinalresearchinstitute.com/tutorials/item-factor-analysis-measurement-invariance-2nd-order-growth-model-ecls-k/. The analytic code and output is available as supplemental material (https://osf.io/hbafn/?view_only=65d1c791a5b74fe7ab71ee0eca56ecdc). Data are not publicly available due to privacy regulations but can be requested for replication, unconditionally and free-of-charge, from TRAILS at www.trails.nl.

All models were estimated with a theta parameterization, pairwise deletion for missing data, a combination of diagonally weighted least squares (DWLS; for parameters) and weighted least square mean and variance adjusted (WLSMV; for robust standard errors) estimation, and nonlinear minimization subject to box constraints (NMLINB) optimization. The first factor loading for each factor was constrained to 1 for identification. Variances and covariances were estimated freely. Latent variable means were constrained to zero and propensity variances for items were constrained to 1 unless otherwise specified below. Items that were in one version of a scale but not the other were still modeled at the appropriate time points to maximize fidelity to clinical use of this measure; however, items that only appeared in one version of the measure were only constrained to equality in different waves of that particular measure. For example, the item “I sleep less than most of my peers” was only assessed in the YSR (i.e., Waves 1–3). As such, the specific equality constraints for testing measurement invariance in this item were only specified in Waves 1 to 3.

As described in the introduction and shown in Figure 1, three types of measurement invariance were tested: configural, metric, and scalar (listed here with increasing stringency). The configural invariance model only imposes the constraint that each item loads onto its specified factor at each time point. The metric invariance (i.e., “weak”) model adds constraints that the factor loadings of an item on its factor are equivalent across timepoints. Finally, the scalar invariance (i.e., “strong”) model incorporates the constraint that item intercepts (in this case thresholds between item-response options) be equivalent across timepoints while latent variable means are allowed to vary. Thus, while the first timepoint for each latent variable mean is set to zero (identical to configural, metric, and scalar invariance models for scaling reasons), latent variable means are estimated freely for later timepoints. There were no additional residual variances because item responses were modeled using thresholds (given ordinal rather than continuous response scales); thus, the scalar invariance model tests both strong and strict invariance. Items that had response options that were not all endorsed at one or more timepoints were dichotomized (“0” = “0” and “1–2” = “1”) at all timepoints to facilitate comparison of item thresholds in that particular sample. The only item this was relevant for was the self-injury item.

Chi-square tests of fit are reported but were not heavily considered regarding conclusions due to over-sensitivity to negligible differences in large sample sizes. Acceptable model fit criteria were a comparative fit index [CFI] ≥ .95, root mean square-error of approximation [RMSEA] ≤ .06, and standardized root-mean-square residual [SRMR] ≤ .08 (Hu et al., 1999). Metric invariance was evaluated based on the following cut-off criteria in change of model fit comparing the metric invariance model to the configural invariance model: −.010 change in CFI, .015 change in RMSEA, and .030 change in SRMR (Chen, 2007). Scalar invariance had identical criteria when comparing the scalar invariance to the metric invariance models except the cut-off for SRMR was reduced to .010 (per Chen, 2007). It is worth noting that these cut-offs were established using continuous data. To our knowledge, cut-offs have not yet been established using ordinal data, and some estimators for ordinal data (including the DWLS/WLSMV used here) have a tendency not to discover misfit (Xia & Yang, 2019). As such, results are preliminary and would benefit from reanalysis when appropriate cut-offs for ordinal data are established.

## Results

Tables 3 includes details about the fit of each model in the population-based sample and the clinic-based sample (respectively). All factor loadings and item thresholds for the models can be found in the supplemental material (https://osf.io/hbafn/?view_only=65d1c791a5b74fe7ab71ee0eca56ecdc).

**Table 3:** Model Fit of Youth and Adult Self-Report Achenbach System of Empirically Based Assessment Scales.

		χ^2^							
Model	*df*	*p*	CFI	ΔCFI	RMSEA	ΔRMSEA	90% CI RMSEA	SRMR	ΔSRMR
Depression/affective problems (*N* = 1,309)									
Configural	3,144	10,139.905 *p* < .001	.958		.041		.040–.042	.075	
Metric	3,205	11,800.126 *p* < .001	.949	−.009	.045	.004	.044–.046	.082	.007
Scalar	3,327	13,858.954 *p* < .001	.937	−.012	.049	.004	.048–.050	.082	.000
Depression/affective problems: Cognitive Subscale (*N* = 1,309)									
Configural	804	2,429.489 *p* < .001	.972		.039		.038–.041	.081	
Metric	834	2,674.661 *p* < .001	.969	−.003	.041	.002	.039–.043	.085	.004
Scalar	894	3,075.272 *p* < .001	.963	−.006	.043	.002	.042–.045	.085	.000
Depression/affective problems: Somatic Subscale (*N* = 1,308)									
Configural	480	2,589.904 *p* < .001	.934		.058		.056–.060	.079	
Metric	502	3,448.137 *p* < .001	.908	−.026	.067	.009	.065–.069	.092	.013
Scalar	551	4,460.389 *p* < .001	.879	−.029	.074	.007	.072–.076	.093	.001
Anxiety (*N* = 1,309)									
Configural	687	2,237.681 *p* < .001	.972		.042		.040–.043	.064	
Metric	712	2,368.522 *p* < .001	.970	−.002	.042	.000	.040–.044	.066	.002
Scalar	767	3,101.770 *p* < .001	.958	−.012	.048	.006	.046–.050	.066	.000
Attention Deficit Hyperactivity Disorder (*N* = 1,309)									
Configural	1,695	7,841.526 *p* < .001	.951		.053		.051–.054	.070	
Metric	1,734	8,755.802 *p* < .001	.943	−.008	.056	.003	.054–.057	.074	.004
Scalar	1,818	9,555.380 *p* < .001	.938	−.005	.057	.001	.056–.058	.074	.000
Somatic (*N* = 1,291)									
Configural	1,065	3,157.095 *p* < .001	.959		.039		.037–.041	.089	
Metric	1,099	3,489.270 *p* < .001	.953	−.006	.041	.002	.040–.043	.093	.004
Scalar	1,172	4,397.557 *p* < .001	.937	−.016	.046	.005	.045–.048	.094	.001

*Note*. Δ = change between current model and previous model (i.e., change between configural and metric and change between metric and scalar). *df* = degrees of freedom; CFI = Comparative Fit Index; RMSEA = root mean square error of approximation; CI = confidence interval; SRMS = standardized root mean square residual.

### Depression Symptoms/Affective Problems Scale

All three interpreted fit indices supported acceptable model fit for the configural invariance model (CFI = .958, RMSEA = .041, and SRMR = .075). Only RMSEA supported acceptable global model fit for the metric model (CFI = .949, RMSEA = .045, SRMR = .082) and the scalar model (CFI = .937, RMSEA = .049, SRMR = .082). All comparisons of model fit supported metric invariance (ΔCFI = −.009, ΔRMSEA = .004, ΔSRMR = .007). Only two out of three comparisons of model fit, specifically ΔRMSEA and ΔSRMR, supported scalar invariance (ΔCFI = −.012, ΔRMSEA = .004, ΔSRMR = .000).

#### Cognitive Symptoms Subscale

Two out of three fit indices (CFI and RMSEA) suggested acceptable model fit for all three invariance models (configural: CFI = .972, RMSEA = .039, SRMR = .081; metric: CFI = .969, RMSEA = .041, SRMR = .085; scalar: CFI = .963, RMSEA = .043, SRMR = .085). All three comparisons of model fit supported metric and scalar invariance of the Cognitive Symptoms Subscale (metric: ΔCFI = −.003, ΔRMSEA = .002, ΔSRMR = .004; scalar: ΔCFI = −.006, ΔRMSEA = .002, ΔSRMR = .000).

#### Somatic Symptoms Subscale

Two out of three fit indices (RMSEA and SRMR) suggested acceptable model fit for the configural invariance model (configural: CFI = .934, RMSEA = .058, SRMR = .079). All model fit indices indicated unacceptable model fit for the metric and scalar models (metric: CFI = .908, RMSEA = .067, SRMR = .092; scalar: CFI = .879, RMSEA = .074, SRMR = .093). Both ΔRMSEA and ΔSRMR supported metric and scalar invariance of the Somatic Symptoms Subscale (metric: ΔRMSEA = .009, ΔSRMR = .013; scalar: ΔRMSEA = .007, ΔSRMR = .001). ΔCFI was the only comparison of model fit that did not support metric and scalar invariance (metric ΔCFI = −.026, scalar ΔCFI = −.029).

### Anxiety Scale

All three interpreted fit indices supported acceptable global model fit for all three invariance models (configural: CFI = .972, RMSEA = .042, and SRMR = .064; metric: CFI = .970, RMSEA = .042, and SRMR = .066; scalar: CFI = .958, RMSEA = .048, and SRMR = .066). All comparisons of model fit supported metric invariance (ΔCFI = −.002, ΔRMSEA = .000, ΔSRMR = .002). Only two out of three comparisons of model fit, specifically ΔRMSEA and ΔSRMR, supported scalar invariance (ΔCFI = −.012, ΔRMSEA = .006, ΔSRMR = .000).

### ADHD Scale

All three interpreted fit indices suggested acceptable global model fit for the configural invariance model (CFI = .951, RMSEA = .053, SRMR = .070). Only two of the three interpreted fit indices (specifically, RMSEA and SRMR) suggested acceptable global model fit for the metric and scalar invariance models (metric: CFI = .943, RMSEA = .056, SRMR = .074; scalar: CFI = .938, RMSEA = .057, SRMR = .074). All three comparisons of model fit supported metric and scalar invariance (metric: ΔCFI = −.008, ΔRMSEA = .003, ΔSRMR = .004; scalar: ΔCFI = −.005, ΔRMSEA = .001, ΔSRMR = .000).

### Somatic Scale

Two of the three interpreted fit indices (CFI and RMSEA) supported acceptable global model fit for the configural and metric invariance models (configural: CFI = .959, RMSEA = .039, and SRMR = .089; metric: CFI = .953, RMSEA = .041, and SRMR = .093). Only RMSEA supported acceptable global fit of the scalar invariance model (CFI = .937, RMSEA = .046, SRMR = .094). All comparisons of model fit supported metric invariance (ΔCFI = −.006, ΔRMSEA = .002, ΔSRMR = .004). Only two out of three comparisons of model fit, specifically ΔRMSEA and ΔSRMR, supported scalar invariance (ΔCFI = −.016, ΔRMSEA = .005, ΔSRMR = .001).

## Discussion

The YSR and ASR are widely used self-report measures of psychological symptoms and well-being. To facilitate their use across developmental periods in research and clinical practice, the YSR and ASR were designed to be comparable measures designed to be developmentally appropriate for youth and adults, respectively. However, the use of these measures to quantify change in symptoms for the same individual requires that they assess psychopathology in the same way across time and across measure forms despite different item wordings to complement intended developmental stages (i.e., YSR→ASR)—otherwise known as longitudinal measurement invariance.

To date, no study has investigated the longitudinal measurement invariance of the Depression/Affective Problems Scale (or its constituent Cognitive and Somatic Subscales), Anxiety Scale, ADHD Scale, and Somatic Scale of the YSR and ASR in a sample where participants completed both measures. This study finds differential support for each of these measures, underscoring the value in separately considering the psychometric properties of multidimensional scales. Results will be discussed in the order they were presented in the Results section (i.e., Depression/Affective Problems, Anxiety, ADHD, and Somatic).

Out of the Depression/Affective Problems Scale, the strongest support was for the Cognitive Symptoms Subscale, which featured consistently good model fit (except the SRMR which was consistently above the cut-off) and all comparisons of model fit indices supported all tested levels of invariance. There was slightly less support for the broader Depression/Affective Problems Scale. Specifically, while all three change indices supported metric invariance, ΔCFI did not support scalar invariance. While the change in model fit statistics is the focal measurement of interest in invariance testing (because it focuses on how model fit reacts to the constraints that define the invariance), it is worth considering that all three absolute model fit statistics (CFI, RMSEA, SRMR) only indicated adequate fit for the configural model. Neither CFI not SRMR supported adequate global fit of the metric or scalar invariance models. There was less support for the global model fit, and longitudinal measurement invariance of, the Somatic Subscale of the Depression/Affective Problems Scale. Two relative change metrics, ΔRMSEA and ΔSRMR, supported the metric and scalar invariance of this scale; however, ΔCFI supported neither metric nor scalar invariance. In fact, the magnitude of the changes in CFI were quite notable (2.6–2.9 × the acceptable cut-off) relative to the other models reported here. With respect to global model fit, only two indices (RMSEA and SRMR) supported acceptable model fit for configural invariance. No global model fit indices supported the metric or scalar invariance models. Consequently, combination use of the Depression/Affective Problems Scale and Cognitive Symptom Subscale of the YSR and ASR are likely suitable for clinical work or research in adolescent and/or adult populations when depression symptoms are of interest; however, the Somatic Symptoms Subscale be used with caution or with adjustments to account for measurement noninvariance (Putnick & Bornstein, 2016).

The Anxiety Scale showed the strongest support for both global model fit and longitudinal measurement invariance across the tested scales, as all metrics supported its psychometric properties except ΔCFI for the scalar invariance model. The ADHD Scale, which had the greatest item-level differences between the YSR and ASR, had consistently strong support for longitudinal measurement invariance (although the CFI was below the cutoff for acceptable model fit in both the metric and scalar invariance models). Finally, all three relative change metrics supported metric invariance and two of the three (ΔRMSEA and ΔSRMR) supported scalar invariance of the Somatic Symptoms Scale. However, it is worth noting that the SRMR was above the cutoff for acceptable global model fit in all three models, and CFI was also below the acceptable cutoff in the scalar invariance model.

One of the key strengths of this study is the inclusion of both the YSR and ASR across multiple time points. Thus, instead of solely testing the longitudinal measurement invariance of one of these measures or using the YSR and ASR in different groups, we were able to evaluate the appropriateness of transitioning from the YSR to the ASR for the same participant or client, as most appropriate for their age. In addition, the sample was large enough to bolster confidence in the generalizability of these findings. Generalizability is further amplified by the fact that this is not an exclusively clinical sample; thus, there are less concerns regarding restriction of range or Berkson’s Bias (Berkson, 1946) than if this study were conducted in a strictly clinical or nonclinical sample. Finally, this study is also the first we are aware of that tested the longitudinal measurement invariance of all of the *DSM-IV* scales shared between the YSR and ASR.

However, this study should also be considered in light of its limitations. First, the self-injury item had to be dichotomized due to lack of participants selecting the most severe option at some waves. Although this is not surprising given the item content relative to the ages of assessment, these are still modeling deviations from standard scoring of the YSR and ASR. Second, as would be expected of most psychiatric symptom data, responses were largely skewed toward less severe responses. Third, meaningful analyses comparing population versus clinical subpopulations were not possible with these data, given lack of information regarding ongoing clinical status in either group.

## Conclusion

In conclusion, this study supports the longitudinal measurement invariance of the YSR and ASR Depression/Affective Problems Scale, Cognitive Symptom Subscale of the Depression/Affective Problems Scale, Anxiety Scale, ADHD Scale, and Somatic Scale. The greatest concerns for longitudinal measurement invariance were for the Somatic Symptoms Subscale of the Depression/Affective Problems Scale. Consequently, clinicians and researchers should carefully consider which items to use, and how to aggregate them, when considering the YSR/ASR as a potential measure to track mental health symptoms overtime and across developmental stages. However, additional work is needed to replicate this study in other samples (e.g., in active episodes of poor mental health) and with different durations between assessments.
